# Risk of urologic cancers in patients with hospital-diagnosed recurrent cystitis: a nationwide population-based study

**DOI:** 10.1038/s41416-026-03591-y

**Published:** 2026-08-19

**Authors:** Kasper Kjersgaard Mortensen, Kirstine Kobberøe Søgaard, Dóra Körmendiné Farkas, Eyal Oren, Mette Nørgaard, Henrik Toft Sørensen

**Affiliations:** 1 Department of Clinical Epidemiology and Center for Population Medicine, Aarhus University and Aarhus University Hospital, Aarhus, Denmark; 2 Department of Clinical Microbiology, Aalborg University Hospital, and Department of Clinical Medicine, Aalborg University, Aalborg, Denmark; 3 Division of Epidemiology and Biostatistics, School of Public Health, San Diego State University, San Diego, CA USA

**Keywords:** Urological cancer, Risk factors, Epidemiology

## Abstract

**Background:**

Recurrent infections in the urinary tract may promote urologic cancer through chronic inflammation. However, evidence linking infections and the subsequent risk of urologic cancer has been conflicting, and long-term risk remains uncertain.

**Methods:**

In this population-based descriptive cohort study, we included all Danish adults with a first-time hospital discharge diagnosis of recurrent cystitis during 2006–2022 in the Danish National Patient Registry, excluding patients with prior cancer. Urologic cancer diagnoses were ascertained through the Danish Cancer Registry. We calculated cumulative incidences and standardised incidence ratios (SIRs) using incidence rates from the general population.

**Results:**

Among 42,377 patients (68% women, median age 62 years), 1047 were diagnosed with urologic cancer (median follow-up 5.7 years). The 1-year risk of any urologic cancer was 1.2% (95% confidence interval [CI], 1.1–1.3%). The corresponding 1-year SIR was 4.99 (95% CI, 4.56–5.44). Beyond 2 years of follow-up, the SIR remained elevated among women only at 1.47 (95% CI, 1.20–1.78).

**Conclusions:**

The elevated short-term risk suggests that recurrent cystitis is a clinical marker of urologic cancer. Yet, with low absolute risks, our results do not suggest additional diagnostic cancer work-up in these patients. In women, long-term risks for urologic cancer remained elevated.

## Introduction

Urinary tract infection (UTI) encompasses infections in the bladder, ureters, and kidneys. Half of all women and nearly 15% of men experience at least one UTI during their lifetime, and recurrence within a year is frequent (approximately 30%-50% for women and 12% for men) [1, 2].

Worldwide, more than 2.5 million people are diagnosed annually with a urologic cancer (urinary bladder, kidney, ureteral and renal pelvic and prostate cancer), and this number is expected to increase as populations age [3]. These cancers are associated with both substantial morbidity and mortality [4–6]. Several studies have shown an elevated risk of urologic cancer among patients with UTIs [7–13].

The development of urologic cancer is multifactorial and shares several risk factors with infections, including smoking and obesity [14–17]. While chronic inflammation from recurrent infections may promote carcinogenesis [18–20], the association between UTI-related inflammation and risk of bladder cancer is not clear [12, 13, 21, 22]. Two meta-analyses, based mainly on case-control studies, reported an association between recurrent UTI (rUTI) and elevated bladder cancer risk, but highlighted substantial between-study heterogeneity and inconsistent control of confounders [23, 24]. Associations between UTI and other urologic cancers have been less extensively studied [8–10, 12, 25–28], with some studies suggesting increased risks of kidney, ureteral, renal pelvic and prostate cancer [8–11]. Recent population-based nationwide studies from Denmark and Sweden have demonstrated a dose-response-dependent association between UTIs and a rare subtype of bladder cancer [29]. The Swedish study further reported a substantially elevated short-term risk of all urologic cancers, as well as a more modest increase in the long-term risks of certain urologic cancers following acute cystitis [30]. The observed short-term risk may, however, partly reflect a misinterpretation of symptoms, as there is a considerable overlap in the clinical presentation of UTIs and urologic cancers [31].

Evidence of long-term risks in patients with a high expected degree of chronic inflammation, such as patients with recurrent UTIs, remains limited and more robust evidence on this potential association may help clinicians develop patient-tailored follow-up regimens, i.e. introducing additional diagnostic work-up for cancer. Because UTI is one of the most common bacterial infections, any association with urologic cancers would have major public-health implications [32].

Historically, varying UTI definitions have complicated interpretation and comparability of studies involving UTI [33]. This challenge also applies to recurrent UTI (rUTI), for which definitions have changed over time [34]. Current guidelines from the European Association of Urology define rUTI as at least three UTI episodes within 1 year or 2 within 6 months [35]. However, no ICD-10 code fully captures this definition in routine registry data. In Denmark, a diagnosis code for recurrent cystitis was introduced in 2004 and captures a clinically relevant subgroup of patients with lower rUTI.

We therefore conducted a nationwide descriptive cohort study among patients with a hospital discharge diagnosis of recurrent cystitis in Denmark. The objective was to describe the absolute risk of urologic cancer and to calculate standardised incidence ratios as a measure of relative risk of urologic cancer compared with the general population.

## Material and methods

### Study design and data collection

We conducted this cohort study within a cumulative source population of approximately 6.2 million people in Denmark from 2006 to 2022. The Danish healthcare system provides tax-funded free medical care to all residents [36]. Vital status and place of residence are tracked by the Danish Civil Register System (CRS). The CRS assigns a unique personal identification number to each Danish resident, which allows linkage of several registries [37]. We defined the study cohort using the Danish National Patient Registry (DNPR), which contains hospital inpatient and outpatient discharge diagnoses coded according to the *International Classification of Diseases*, *Tenth Revision* (ICD-10). Each contact includes one primary diagnosis code and may include several secondary diagnosis codes [38]. The DNPR includes data from all Danish public hospitals and private hospitals but does not capture information from general practice.

We identified all patients aged ≥18 years in the DNPR with a first-time hospital-based diagnosis code of recurrent cystitis (inpatient or outpatient contact, primary or secondary diagnosis codes) between 1 January 2006 and 31 December 2022. The index date was defined as the date of hospital admission or first outpatient contact associated with a recurrent cystitis diagnosis. We excluded patients with a recurrent cystitis diagnosis before 2006 to reduce misclassification due to variability in coding practices during the early implementation of the recurrent cystitis code (introduced in 2004).

### Cancer

Incident cancers in Denmark are recorded in the Danish Cancer Registry (DCR) since 1943, with high specificity and data completeness [39]. We used the DCR to identify all incident urologic cancers (urinary bladder, kidney, ureteral and renal pelvic, and prostate cancer) registered during follow-up. We excluded patients with any prior cancer (except non-malignant skin cancer [NMSC]) before the recurrent cystitis diagnosis.

### Covariates

Overall comorbidity was characterised using a modified Charlson Comorbidity Index (CCI) score (Table S1), which did not include cancer due to the exclusion of patients with prior cancer [40, 41]. We included diagnoses recorded within 10 years prior to the index date. The CCI score was categorised into the following groups: 0 (low), 1–2 (moderate) and ≥3 (severe).

For selected comorbidities (benign prostatic hyperplasia, gynaecological diseases, diabetes, hypertension, chronic kidney disease, obesity, chronic obstructive pulmonary disease [COPD] and a history of urinary tract stone), we used a 10-year lookback period from the index date to enhance sensitivity while ensuring clinically relevant diagnoses.

We used a 1-year lookback period for a hospital discharge diagnosis of haematuria, as this can be a presenting sign of cancer. We identified the department’s main speciality that assigned the recurrent cystitis diagnosis, using information in the DNPR.

To give an overview of the diagnostic work-up of patients with recurrent cystitis, we included information on abdominal and/or urinary tract computed tomography (CT) scans, or other urinary tract–related procedures and tests, performed within 30 days before or after the index date.

To characterise the burden of acute infections in the urinary tract, we assessed the number of UTI episodes in the year preceding the index date. We defined an episode of UTI as either a hospital discharge diagnosis of UTI or a redeemed prescription for a UTI-specific antibiotic. Subsequent diagnoses or prescriptions redeemed within 14 days were considered part of the same episode. We used the Danish National Prescription Registry to identify redeemed prescriptions. This registry contains complete and valid information on prescriptions dispensed at Danish community pharmacies since 1995, including dispensing dates and Anatomical Therapeutic Chemical (ATC) codes [42]. With the available data, it was not possible to determine whether a prescribed antibiotic was specifically used to treat cystitis; therefore, the analysis was limited to acute UTIs. All ATC and ICD-10 codes are listed in Supplementary Table S2.

### Statistical analysis

We characterised the cohort at the index date according to sex, median age with interquartile range (IQR), age categories (18–59, 60–74, ≥75 years), modified CCI score, selected comorbidities, and other baseline characteristics. Categorical variables were reported as total numbers with proportions (n, %). We followed patients from the index date until a diagnosis of urologic cancer, emigration, death, or end of the study period (31 December 2022), whichever came first. We calculated the median follow-up time with IQR.

The absolute risk was calculated as cumulative incidences of cancer after 1, 2 and 5 years, treating death as a competing risk [43]. For prostate cancer, the total number of men in the cohort was used as the denominator in calculating absolute risks. For other cancers, cumulative incidences were based on the entire cohort.

We compared the observed number of urologic cancers among patients with a hospital discharge diagnosis of recurrent cystitis with the number expected in the general Danish population. Expected cancer counts were estimated using national cancer incidence rates stratified by age, sex and calendar year. We calculated standardised incidence ratios (SIRs) as a measure of relative risk. Follow-up was categorised into 0- < 1 years (short-term), 1- < 2 years, ≥2 years (long-term) and total follow-up time. We calculated SIRs for urologic cancer for patients in the recurrent cystitis cohort categorised by sex, age, type of diagnosis (primary or secondary), speciality (urology or non-urology), modified CCI score, number of acute UTIs, presence of haematuria and history of urinary tract stones. For selected comorbidities, we performed subgroup analyses to explore potential shared risk factors.

To contextualise the absolute risk of urologic cancer in a clinically interpretable manner, we calculated the number of patients needed to undergo diagnostic evaluation to detect one urological cancer (not excess cancer) as the reciprocal of the absolute risk. This calculation assumes that cancers diagnosed within 1 year after the recurrent cystitis diagnosis were already present at the time of that diagnosis, and that the diagnostic work-up would identify all occult cancers. We estimated the number-needed-to-examine separately in both sexes for urinary bladder cancer and kidney cancer, and among men for prostate cancer. Due to the low number of  ureteral and renal pelvic cancers, the number-needed-to-examine was not calculated for these cancer types.

To compute 95% confidence intervals (CIs), we used exact limits when the observed number of cases was less than 10; otherwise, we used Byar’s approximation [44], assuming the observed number of cancers followed a Poisson distribution.

SAS version 9.4 (SAS Institute, Cary, NC) was used for data management and statistical analyses.

A Large Language Model (Chat-GPT) was used to improve language and readability. The output was reviewed and edited as needed, and the authors take full responsibility for the content of the published article.

### Ethics

The study was reported to the Danish Data Protection Agency by Aarhus University (Serial number 2016-051-000001/812). Under Danish legislation, registry-based studies based exclusively on existing administrative and health registry data do not require approval from a research ethics committee or informed consent from participants. The study was conducted in accordance with the Declaration of Helsinki [45].

## Results

### Study cohort

We identified 42,377 patients with a first-time hospital discharge diagnosis of recurrent cystitis between 2006 and 2022 (Table 1). Women accounted for 68% of the cohort. Compared with men, women were generally younger, and more often received a diagnosis of recurrent cystitis diagnoses outside a urology department (46% vs. 30%). The overall median age was 62 years (IQR 44-73 years), and most patients had a primary diagnosis code for recurrent cystitis (84% of women and 83% of men). Among men, 14% had a hospital discharge diagnosis of benign prostatic hyperplasia, and 18% of women had a diagnosis of gynaecological disease. Two-thirds of patients (66%) had no record of comorbidities included in the modified CCI, with proportions 69% among women, and 59% among men. Overall, 21% had hypertension, 7% had chronic kidney disease, and 5% of the patients had COPD. A lower proportion of women had diabetes compared with men (8% vs. 13%), whereas obesity was more common among women (9% vs. 4%). Almost all patients (94%) experienced at least one UTI episode within 1 year before the index date, and 57% (63% and 45% among women and men) had three or more episodes (Table 1). Most episodes were managed in general practice, with only 14% of patients having a hospital discharge diagnosis of UTI.

**Table 1:** Characteristics of patients with a first-time diagnosis of recurrent cystitis in Denmark, 2006–2022.

	Women, *n* (%)	Men, *n* (%)	Overall, *n* (%)
All patients	28,705 (100.0)	13,672 (100.0)	42,377 (100.0)
Age in years, median (IQR)	57.5 (37.8–72.2)	66.6 (55.8–74.9)	61.7 (44.0–73.3)
Age group			
18–59	15,397 (53.6)	4510 (33.0)	19,907 (47.0)
60–74	7652 (26.7)	5798 (42.4)	13,450 (31.7)
≥75	5656 (19.7)	3364 (24.6)	9020 (21.3)
Type of diagnosis			
Primary	24,055 (83.8)	11,268 (82.4)	35,323 (83.4)
Secondary	4650 (16.2)	2404 (17.6)	7054 (16.6)
Specialty			
Urology	15,594 (54.3)	9569 (70.0)	25,163 (59.4)
Non-urology	13,111 (45.7)	4103 (30.0)	17,214 (40.6)
Comorbidities^a^			
Diabetes	2248 (7.8)	1722 (12.6)	3970 (9.4)
Hypertension	5695 (19.8)	3263 (23.9)	8958 (21.1)
Chronic kidney disease	1820 (6.3)	1178 (8.6)	2998 (7.1)
Obesity	2661 (9.3)	555 (4.1)	3216 (7.6)
Chronic obstructive pulmonary disease	1339 (4.7)	763 (5.6)	2102 (5.0)
Benign prostatic hyperplasia^b^	-	1878 (13.7)	1878 (4.4)
Gynaecological diseases^c^	5249 (18.3)	-	5249 (12.4)
Modified CCI score^d^			
0	19,897 (69.3)	8069 (59.0)	27,966 (66.0)
1–2	7031 (24.5)	4072 (29.8)	11,103 (26.2)
≥3	1777 (6.2)	1531 (11.2)	3308 (7.8)
Number of UTI episodes^e^			
0	1521 (5.3)	1000 (7.3)	2521 (5.9)
≥1	27,184 (94.7)	12,672 (92.7)	39,856 (94.1)
1–2	8983 (31.3)	6539 (47.8)	15,522 (36.6)
3–5	13,192 (46.0)	4712 (34.5)	17,904 (42.2)
≥6	5009 (17.4)	1421 (10.4)	6430 (15.2)
Heamaturia^e^	604 (2.1)	508 (3.7)	1112 (2.6)
Urinary tract stone^a^	1275 (4.4)	862 (6.3)	2137 (5.0)
CT scans^f^			
Abdomen and pelvis	6771 (23.6)	3740 (27.4)	10,511 (24.8)
Kidney and urinary tract	1249 (4.4)	706 (5.2)	1955 (4.6)
Urography	4973 (17.3)	2720 (19.9)	7693 (18.2)
Procedures and tests^f^			
Cystoscopy	3058 (10.7)	1902 (13.9)	4960 (11.7)
Uroflowmetry	12,603 (43.9)	7,381 (54.0)	19,984 (47.2)
Ultrasound for residual urine	6740 (23.5)	3229 (23.6)	9969 (23.5)
Fluid and urination schedule	1660 (5.8)	819 (6.0)	2479 (5.8)

*IQR* interquartile range, *CT* computerised tomography, *CCI* Charlson Comorbidity Index, *UTI* urinary tract infection.

^a^Look-back period of 10 years from recurrent cystitis diagnosis.

^b^Among men only.

^c^Among women only.

^d^Excluding cancers.

^e^Look-back period of 1 year from recurrent cystitis diagnosis.

^f^±30 days from recurrent cystitis diagnosis.

### Procedures

In the period 30 days before to 30 days after the index date, almost half of the patients diagnosed with recurrent cystitis underwent a uroflowmetry, 25% had a CT scan of the abdomen and pelvis (including urography), and 12% were evaluated with cystoscopy (Table 1). Overall, approximately a third of patients had a CT scan and/or a cystoscopy (30% of women, 36% of men).

### Risk of any urologic cancer

During a median follow-up of 5.7 years (IQR 2.7–9.1 years), we observed 1047 urologic cancers, including 431 urinary bladder cancers, 122 kidney cancers, 34 ureteral and renal pelvic cancers, and 460 prostate cancers.

The 1- and 2-year absolute risks of any cancer were 1.2% (95% CI, 1.1%-1.3%) and 1.5% (95% CI, 1.4%-1.6%), respectively (Table 2). At 5 years of follow-up, the absolute risk of any urologic cancer cumulated to 2.2% (95% CI, 2.0–2.3%). Men had a higher risk of being diagnosed with an incident urologic cancer compared to women, even after excluding prostate cancers. The risk of urologic cancers increased with age, with a higher risk in patients over the age of 60 years compared to those aged 18–59 years (Table 2).

**Table 2:** Cumulative incidence (%, 95% CI) of urologic cancer in patients with a first-time diagnosis of recurrent cystitis in Denmark, 2006–2022.

	1-year incidence	2-year incidence	5-year incidence
Any urologic cancer	1.2 (1.1–1.3)	1.5 (1.4–1.6)	2.2 (2.0–2.3)
Age group			
18–59 years	0.4 (0.3–0.5)	0.5 (0.4–0.6)	0.7 (0.6–0.9)
60–74 years	1.9 (1.7–2.2)	2.3 (2.0–2.5)	3.5 (3.2–3.8)
≥75 years	1.9 (1.6–2.2)	2.5 (2.2–2.8)	3.4 (3.0–3.8)
Sex			
Women	0.6 (0.5–0.7)	0.7 (0.6–0.8)	0.9 (0.8–1.0)
Men	2.5 (2.3–2.8)	3.1 (2.8–3.4)	4.9 (4.5–5.3)
Any urologic cancer minus prostate cancer	0.7 (0.6–0.8)	0.9 (0.8–1.0)	1.2 (1.1–1.3)
Men	1.0 (0.9–1.2)	1.3 (1.1–1.5)	1.8 (1.6–2.1)
Urologic cancers			
Urinary bladder	0.6 (0.5–0.7)	0.7 (0.6–0.8)	0.9 (0.8–1.0)
Kidney	0.1 (0.1–0.1)	0.1 (0.1–0.2)	0.2 (0.2–0.3)
Ureter and renal pelvis	0.0 (0.0–0.0)	0.0 (0.0–0.1)	0.1 (0.0–0.1)
Prostate^a^	1.5 (1.3–1.7)	1.8 (1.6–2.1)	3.1 (2.8–3.4)

*CI* Confidence Interval.

^a^Calculated among men only.

**Fig. 1:**
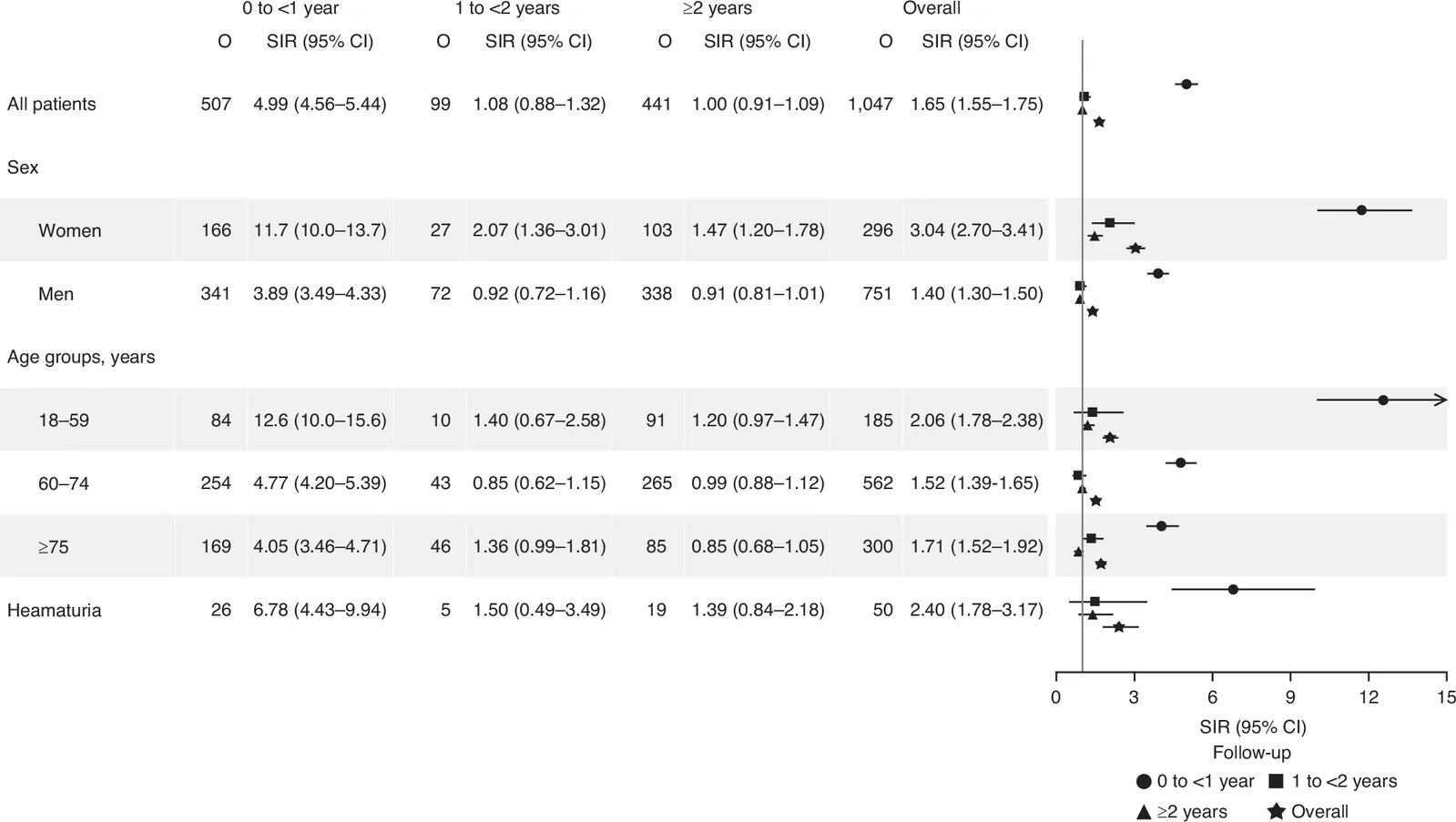
Observed number, and standardised incidence ratios of urologic cancer following a first-time diagnosis of recurrent cystitis in Denmark, 2006–2022, according to sex, age, haematuria and follow-up time. Abbreviations: O observed cancers, SIR standardised incidence ratio, CI confidence interval.

The SIR for urologic cancer for the entire follow-up period was 1.65 (95% CI, 1.55–1.75) (Fig. 1). Within 1 year of follow-up, the overall SIR for any urologic cancer was 4.99 (95% CI, 4.56–5.44). The overall SIR decreased to 1.08 (95% CI, 0.88–1.32) and 1.00 (95% CI, 0.91–1.09) for 1- < 2 and ≥2 years of follow-up, respectively. Among women, the corresponding SIRs remained elevated at 2.07 (95% CI, 1.36–3.01) and 1.47 (95% CI, 1.20–1.78), respectively (Fig. 1).

### Subgroup analyses

**Fig. 2:**
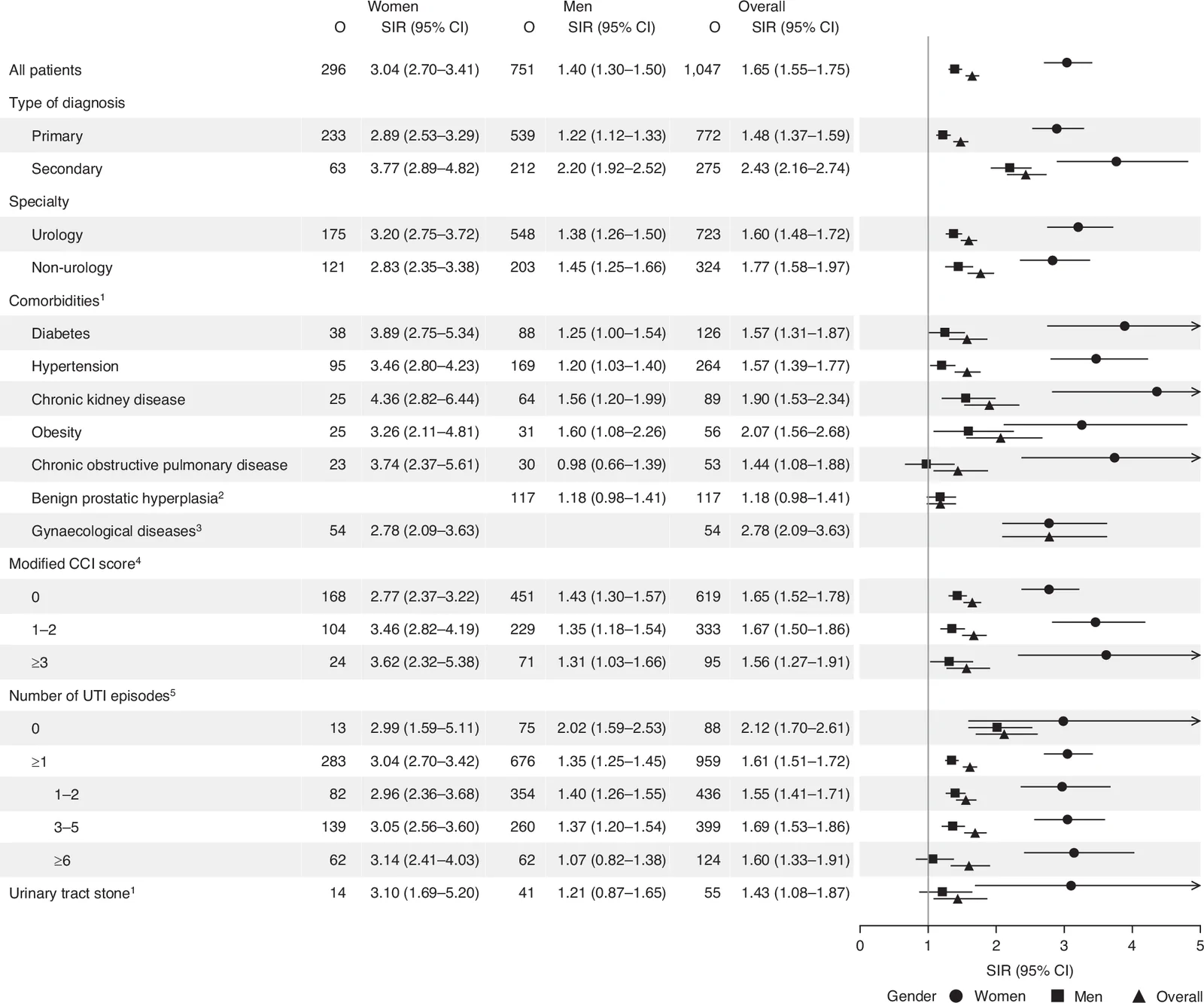
Observed number, and standardised incidence ratios of urologic cancers in patients with a first-time diagnosis of recurrent cystitis in Denmark, 2006–2022. ^1^Look-back period of 10 years from recurrent cystitis diagnosis. ^2^Among men only. ^3^Among women only. ^4^Excluding cancers. ^5^Look-back period of 1 year from recurrent cystitis diagnosis. Abbreviations: O observed cancers, CCI Charlson Comorbidity Index, CI confidence interval.

Men with benign prostatic hyperplasia had an absolute risk of urologic cancer within 1 year of 1.8% (95% CI, 1.3–2.5%), rising to 2.8% (95% CI, 2.1–3.6%) and 5.6 (95% CI, 4.6–6.8%) after 2 and 5 years, respectively (data not shown). The overall SIR for any urologic cancer in this group was 1.18 (95% CI, 0.98–1.41) (Fig. 2).

For women with gynaecological diseases, the 1-, 2- and 5-year absolute risks were comparable to those of the overall female cohort (data not shown). The overall SIR was 2.78 (95% CI, 2.09–3.63) (Fig. 2).

Patients with obesity had a twofold increase in the overall risk of a urologic cancer diagnosis compared with the general population (SIR = 2.07 [95% CI, 1.56–2.68]) (Fig. 2). For patients with hypertension, COPD, or diabetes, the relative risk of urologic cancer was approximately 50% higher than that of the general population. Patients with one or more UTI episodes within a year before the index date also had an elevated risk of urologic cancer, with a SIR of 1.61 (95% CI, 1.51–1.72) (Fig. 2). Across all sex-stratified subgroup analyses, the highest overall SIRs were observed among women.

Only 1112 (3%) patients had a diagnosis code of haematuria within 1 year before the index date. Among these patients, the 1-year SIR for any urologic cancer was 6.78 (95% CI, 4.43–9.94), and the SIR beyond 2 years of follow-up was 1.39 (95% CI, 0.84–2.18) (Fig. 1).

### Risk of urinary bladder cancer

The absolute risk of bladder cancer within 2 years of follow-up was 0.9% (95% CI, 0.8–1.0%) (Table 2). Sex-specific 2-year risks were 0.5% (95% CI, 0.5–0.6%) for women and 1.0% (95% CI, 0.9–1.2%) for men, respectively (data not shown). The relative risk of bladder cancer within 1 year of follow-up was almost ninefold higher among patients with recurrent cystitis compared with the general population (Table 3). Women had a 40% increase in the risk of bladder cancer at more than 2 years of follow-up compared with the general population (SIR = 1.40 [95% CI, 1.07–1.81]). Among patients aged 18–59, the SIR was elevated at 1.43 (95% CI, 0.95–2.06) with more than 2 years of follow-up (Table 3).

**Table 3:** Observed numbers, and standardised incidence ratios of urologic cancer following a first-time diagnosis of recurrent cystitis in Denmark, 2006–2022, according to type of cancer, age, sex and follow-up time.

	0 - <1 year		1 - <2 years		≥2 years		Overall	
	O	SIR (95% CI)	O	SIR (95% CI)	O	SIR (95% CI)	O	SIR (95% CI)
Urologic cancer, any	507	4.99 (4.56–5.44)	99	1.08 (0.88–1.32)	441	1.00 (0.91–1.09)	1047	1.65 (1.55–1.75)
Urinary bladder	249	8.68 (7.64–9.83)	40	1.54 (1.10–2.10)	142	1.11 (0.93–1.30)	431	2.35 (2.14–2.59)
Sex								
Women	137	15.96 (13.40–18.87)	15	1.90 (1.06–3.13)	59	1.40 (1.07–1.81)	211	3.60 (3.13–4.12)
Men	112	5.57 (4.59–6.71)	25	1.39 (0.90–2.05)	83	0.96 (0.77–1.19)	220	1.77 (1.54–2.02)
Age group, years								
18–59	48	25.72 (18.96–34.10)	6	3.06 (1.12–6.66)	28	1.43 (0.95–2.06)	82	3.49 (2.78–4.34)
60–74	107	8.44 (6.91–10.20)	12	0.98 (0.51–1.72)	77	1.08 (0.85–1.35)	196	2.03 (1.76–2.34)
≥ 75	94	6.65 (5.38–8.14)	22	1.87 (1.17–2.82)	37	0.99 (0.70–1.37)	153	2.42 (2.05–2.83)
Kidney	43	3.84 (2.78–5.18)	13	1.27 (0.68–2.17)	66	1.24 (0.96–1.58)	122	1.64 (1.36–1.96)
Sex								
Women	*	4.29 (2.62–6.63)	*	2.08 (0.95–3.95)	*	1.39 (0.96–1.95)	62	1.90 (1.45–2.43)
Men	*	3.52 (2.23–5.29)	<5	N/A	*	1.13 (0.77–1.58)	60	1.44 (1.10–1.85)
Age group, years								
18–59	*	7.30 (3.77–12.75)	<5	N/A	*	0.95 (0.52–1.59)	28	1.54 (1.03–2.23)
60–74	*	3.95 (2.50–5.93)	*	0.91 (0.29–2.11)	*	1.52 (1.11–2.04)	73	1.79 (1.40–2.25)
≥75	*	2.15 (0.93–4.23)	*	1.97 (0.72–4.30)	*	0.81 (0.32–1.66)	21	1.36 (0.84–2.08)
Ureter and renal pelvis	11	6.11 (3.05–10.93)	6	3.68 (1.35–8.02)	17	2.10 (1.22–3.36)	34	2.95 (2.04–4.12)
Sex								
Women	*	10.12 (4.64–19.24)	<5	N/A	*	2.54 (1.27–4.55)	23	3.81 (2.42–5.72)
Men	<5	N/A	<5	N/A	*	1.59 (0.58–3.47)	11	2.00 (1.00–3.58)
Age group, years								
18–59	<5	N/A	<5	N/A	<5	N/A	5	3.75 (1.22–8.74)
60–74	*	7.78 (2.85–16.95)	<5	N/A	*	2.24 (1.07–4.12)	19	3.18 (1.91–4.97)
≥75	<5	N/A	<5	N/A	<5	N/A	10	2.37 (1.13–4.36)
Prostate	204	3.40 (2.95–3.90)	40	0.75 (0.53–1.02)	216	0.85 (0.74–0.98)	460	1.26 (1.14–1.38)
18–59 years	23	7.49 (4.75–11.24)	<5	N/A	*	1.12 (0.81–1.49)	70	1.50 (1.17–1.89)
60–74 years	118	3.47 (2.87–4.16)	*	0.72 (0.46–1.08)	*	0.82 (0.69–0.98)	274	1.21 (1.07–1.36)
≥ 75 years	63	2.74 (2.11–3.51)	15	0.82 (0.46–1.36)	38	0.74 (0.53–1.02)	116	1.25 (1.04–1.51)

*Observed number of cancers not reported according to Danish data protection rules to prevent back-calculation of cells with less than 5 observations.

*O* observed cancers, *SIR* Standardised incidence ratio, CI confidence interval, N/A not applicable.

### Risk of kidney, ureteral and renal pelvic cancer

The absolute 2-year risks of kidney cancer and cancer of the ureter and renal pelvis were low at 0.1% (95% CI, 0.1–0.2%) and 0.0% (0.0–0.1%), respectively (Table 2). The overall SIRs at 1-year follow-up were elevated substantially at 3.84 (95% CI, 2.79–5.18) for kidney cancer, and at 6.11 (95% CI, 3.05-10.93) for ureteral and renal pelvic cancer. Beyond 2 years of follow-up, the overall SIRs decreased to 1.24 (95% CI, 0.96–1.58) and 2.10 (95% CI, 1.22–3.36), respectively. This elevated SIR for ureteral and renal pelvic cancer was most attenuated among women, with a SIR of 2.54 (95% CI, 1.27–4.55). The SIR for kidney cancer beyond 2 years of follow-up was 1.13 (95% CI, 0.77–1.58) for men and 1.39 (95% CI, 0.96–1.95) for women (Table 3).

### Risk of prostate cancer

Almost half of the urologic cancers observed in men were prostate cancers, with a 2- and 5-year risk of 1.8% (95% CI, 1.6–2.1%) and 3.1% (95% CI, 2.8–3.4%), respectively (Table 2). The SIR for prostate cancer was elevated with 1-year follow-up at 3.40 (95% CI, 2.95–3.90). No increased risk was observed beyond 1 year of follow-up (Table 3).

### Number-needed-to-examine

The number-needed-to-examine to detect one urologic cancer within 1 year following a diagnosis of recurrent cystitis varied by cancer type and sex. The estimates are based on the assumptions that all cancers diagnosed within 1 year of the index date, were present at that time, and that the diagnostic work-up would detect all cancers. For bladder and kidney cancer, the overall numbers-needed-to-examine were 168 (95% CI, 149–190) and 969 (95% CI, 723–1315), respectively. Among women, 207 (95% CI, 176–245) would need to be examined to detect one urinary bladder cancer within 1 year of follow-up compared with 120 (95% CI, 100–145) among men. To detect one kidney cancer, 1413 (95% CI, 923–2219) women and 583 (95% CI, 393–890) men would need to undergo diagnostic evaluation. For prostate cancer, the number-needed-to-examine was approximately 66 (95% CI, 58–76).

## Discussion

In this population-based cohort, we found that the 1-year relative risk of any urologic cancer was substantially elevated and that risks beyond 2 years of follow-up generally were low but varied by sex.

There may be several explanations for the elevated relative risks observed during the first year of follow-up. They could reflect that cancers were detected during the diagnostic evaluation for recurrent cystitis. Although the SIRs declined after the first year of follow-up, no compensatory deficit in cancer risk was observed. This suggests that detection bias alone may not fully account for this elevated short-term risk and that recurrent cystitis could be a symptom of occult urologic cancer. This interpretation aligns with the findings by Funston et al., who reported elevated rates of prescriptions for UTI-specific antibiotics up to 9 months before bladder and kidney cancer diagnosis, indicating the potential for an earlier cancer diagnosis among patients presenting with UTI symptoms [46]. Several possible explanations exist to support these findings. Urologic cancers, or even precancerous lesions, may increase the risk of UTI by directly affecting the urological epithelium or by compromising host defence [47], and urologic cancers may present with symptoms similar to UTI [31].

When focusing on outcomes beyond 2 years of follow-up, women exhibited consistently elevated relative risks across all urologic cancers, including urinary bladder cancer. This contrasts with findings from a recent Danish case–control study based on redeemed prescriptions for UTI-specific antibiotics commonly used to treat cystitis [29]. That study found no association between multiple UTIs and urothelial carcinoma of the bladder but reported a dose–response association with squamous cell carcinoma, applying a 2-year lag time to account for potential reverse causation. Methodological differences may explain this contrast. Pottegård et al. included community-treated UTIs, whereas our study focused on hospital discharge diagnoses of recurrent cystitis [29]. Furthermore, our study did not distinguish between histological subtypes. However, with urothelial carcinomas being the most prevalent subtype [48], we believe these accounted for most urinary bladder cancer cases in our study. Finally, Pottegård et al. did not stratify their main analysis by sex, leaving potential disparities according to sex unexplored [29].

Our observation of elevated long-term relative risks in women aligns with findings from a nationwide Swedish cohort study, which included patients with first-time acute cystitis [30]. They found increased 5-year SIRs among women (bladder cancer: 1.69 [95% CI 1.58–1.82], kidney cancer: 1.38 [95% CI, 1.25–1.51]). In contrast to our study, they also described an increased risk among men at 5 years of follow-up, but for bladder cancer only (SIR = 1.53 [95% CI, 1.43–1.64]). It is important to note several methodological differences between our studies. Besides including patients with acute cystitis, Jansåker et al. included data from primary health care, adjusted for sociodemographic and comorbidities, and applied longer and broader follow-up periods [30]. Despite these differences, we believe both studies emphasise a long-term elevated risk of urological cancer after cystitis in women.

Studies exploring this sex disparity highlighted several possible explanations. The long-term elevated risks in women may reflect delayed diagnosis, potentially arising from different approaches to the diagnostic evaluation between men and women [31, 46, 49]. Furthermore, a systematic review concluded that women with haematuria were more likely than men to receive treatment for UTI or pain and that women didn’t receive the same level of diagnostic workup as compared to their male counterparts [50].

The very low absolute risks, especially for urinary bladder, kidney and ureteral and renal pelvic cancer, put our relative estimates into perspective. Low absolute risks have previously been reported in Denmark in patients above 50 years of age with pyelonephritis [7]. The low risks presented in our study resulted in a very high number-needed-to-examine to diagnose one urologic cancer ranging from more than 1400 for kidney cancer in women, to less than 70 for prostate cancer in men. The lower number-needed-to-examine to detect prostate cancer does not necessarily translate into a net clinical benefit. It may primarily increase the detection of indolent cancers that would never require treatment, thereby exposing men to the harms of overtreatment [51]. Furthermore, the number-needed-to-examine are estimated under assumptions that may not fully reflect clinical conditions and are likely underestimated. Improved stratification tools could potentially reduce these numbers in the future, but they currently do not justify additional diagnostic work-up in patients with recurrent cystitis.

### Strengths and limitations

In our population-based approach, we have virtually complete data on the study population and minimal loss to follow-up. Several limitations should be considered. We only included patients diagnosed with recurrent cystitis at the hospital. Less complicated cases are treated in the primary care setting, and our results should be applied cautiously to this group. However, it is difficult to speculate if patients diagnosed with recurrent cystitis at the hospital have a higher or lower cancer risk than patients diagnosed in primary care only. A priori, patients seen at the hospital may have a higher cancer risk, yet high-risk individuals are typically enroled through their general practitioner in Cancer Patient Pathways [52] that provide a fast-track diagnostic work-up for cancer.

We cannot rule out diagnostic misclassification of either UTI episodes or the recurrent cystitis diagnosis. Misclassification of the latter may in part reflect historical inconsistencies in the definition of rUTI [34]. Furthermore, the ICD-10 code for recurrent cystitis was introduced in Denmark in 2004, and its use has likely evolved over time, potentially affecting our estimates in unpredictable ways despite the exclusion of patients diagnosed before 2006. Additionally, as the ICD-10 coding for recurrent cystitis has not been validated, its positive and negative predictive values remain unknown.

A total of 5.9% of patients did not have any documented UTIs in the year prior to the index date and did not meet the criteria for rUTI according to the definition by the European Association of Urology. However, the number of UTIs was defined based on specific UTI-related ICD-codes and redeemed prescriptions for UTI-specific antibiotics, both of which may underestimate the true number of infections, especially when non-specific antibiotics are used. The diagnosis of recurrent cystitis is based on an overall clinical assessment, and not all relevant information is captured in registry data. When restricting the analysis to patients with at least one documented UTI, the results remained unchanged (Supplementary Table S3–S5).

Finally, unmeasured variables may have influenced the risk of urologic cancer. We lacked data on lifestyle factors, and our methodological approach did not allow for standardisation beyond age, sex and year of recurrent cystitis diagnosis. Shared risk factors such as smoking and obesity may indicate a common underlying cause of both recurrent cystitis and cancer and may partly explain the elevated relative risk estimates observed among patients with recurrent cystitis compared with the general population.

In conclusion, the elevated short-term relative risks suggest that recurrent cystitis may be a clinical marker of urologic cancer. However, the absolute risks of urologic cancer in these patients remain low, and our findings do not support the need for additional cancer-related diagnostic investigations. In women, however, the long-term risks of urologic cancer remained elevated. Several explanations are possible, including delayed diagnosis of an underlying urologic cancer, shared risk factors or a potential causal association between recurrent cystitis and the development of urologic cancer.

## Supplementary information

Supplemental Material

STROBE checklist

## Data Availability

In Denmark, registry data are kept at secured research servers and data sharing is not permitted according to Danish legislation.
