# Supplementary material for: Risk of urologic cancers in patients with hospital-diagnosed recurrent cystitis: a nationwide population-based study
Source: Br J Cancer. 2026 Aug 19;135(10):1666–74. doi: 10.1038/s41416-026-03591-y (PMC13740257; doi:10.1038/s41416-026-03591-y)
Supplement: Supplementary file 1 [file 41416_2026_3591_MOESM1_ESM.pdf]

## Supplemental Material

Table S1. Charlson Comorbidity Index conditions.

Primary/secondary diagnosis codes from non-emergency departments during 10 years before the index date

| Disease | ICD-10 codes | Score |
|---|---|---|
| Myocardial infarction | I21, I22, I23 | 1 |
| Congestive heart failure | I50, I110, I130, I132, | 1 |
| Peripheral vascular disease | I70, I71, I72, I73, I74, I77, | 1 |
| Cerebrovascular disease | I60, I61, I62, I63, I64, I65, I66, I67, I68, I69, G45, G46 | 1 |
| Dementia | F00, F01, F02, F03, F051, G30 | 1 |
| Chronic pulmonary disease | J40, J41, J42, J43, J44, J45, J46, J47, J60, J61, J62, J63, J64, J65, J66, J67, J684, J701, J703, J841, J920, J961, J982, J983 | 1 |
| Connective tissue disease | M05, M06, M08, M09, M30, M31, M32, M33, M34, M35, M36, D86 | 1 |
| Ulcer disease | K221, K25, K26, K27, K28, | 1 |
| Mild liver disease | B18, K700, K701, K702, K703, K709, K71, K73, K74, K760 | 1 |
| Diabetes type1<br>Diabetes type2 | E100, E101, E109<br>E110, E111, E119 | 1 |
| Hemiplegia | G81, G82 | 2 |
| Moderate to severe renal disease | I12, I13, N00, N01, N02, N03, N04, N05, N07, N11, N14, N17, N18, N19, Q61 | 2 |
| Diabetes with end-organ damage<br>type1<br>type2 | <br>E102, E103, E104, E105, E106, E107, E108<br>E112, E113, E114, E115, E116, E117, E118 | 2 |
| Any tumor | C00-C75 | EXCLUDED |
| Leukemia | C91, C92, C93, C94, C95 | EXCLUDED |
| Lymphoma | C81, C82, C83, C84, C85, C88, C90, C96 | EXCLUDED |
| Moderate to severe liver disease | B150, B160, B162, B190, K704, K72, K766, I85 | 3 |
| Metastatic solid tumor | C76, C77, C78, C79, C80 | EXCLUDED |
| AIDS | B21, B22, B23, B24 | 6 |

Table S2. Codes (diagnosis, treatment, procedure, ATC)

| Disease | ICD-10 / treatment / procedure codes | ATC codes |
|---|---|---|
| Recurrent cystitis | N308C | |
| Events within 1 year before index date (primary/secondary diagnosis codes from all departments) | | |
| UTI episode<br>*An episode is defined by any of the relevant ICD codes or a redeemed prescription 14 days apart* | N300, N309, N390, N109, N129C, N209A, O230, O862B | J01EA01, J01EB02, J01CA08, J01XE01 |
| Haematuria | R319, N029A, N029B | |
| Comorbidities (primary/secondary diagnosis codes from non-emergency department during 10 years before index date) | | |
| Benign prostatic hyperplasia | N40 | |
| Gynaecological diseases | N70, N71, N72, N73, N74, N75, N76, N77, N80, N81, N82, N83, N84, N85, N86, N87, N88, N89, N90 | |
| Chronic obstructive pulmonary disease (COPD) | J44 | |
| Hypertension | I10, I11, I12, I13, I14, I15 | |
| Diabetes | E10, E11, E12, E13, E14, O240, O241, O242, O243, O245, O249, G632, H360, N083 | |
| Obesity | E66 | |
| Chronic kidney disease | E102, E112, E122, E132, E142, G62, I12, I13, N02, N03, N07, N083, N11, N14, N150, N16, N18, N19, Q60, Q61, Q62, Q63, Q64, T817E1, T861, Z940, Z992, KKAS, BJFD0, BJFD2 | |
| Urinary tract stones | N20, N21, N22, N23, N132 | |
| Any prior cancer identified from the DNPR | | |
| Any cancer | C00-C96 (except C44) | |
| Urologic cancers identified from the DCR | | |
| Urologic cancers | | |
| - Urinary bladder cancer | C67, D090, D095, D303, D414 (used together with morphology codes 812-813) | |
| - Kidney cancer | C64 | |
| - Ureteral and renal pelvic cancer | C65, C66, D301, D411, D302, D412 (used together with morphology codes 812-813) | |
| - Prostate cancer | C61 | |
| CT scan (30 days before and after index date) | | |
| Abdomen | UXCD | |
| Kidney and urinary tract | UXCD60 | |
| Kidney | UXCD61 | |
| Urography | UXCD62 | |

| Procedures (30 days before and after index date) | | |
|---|---|---|
| Cystoscopy | KUKC, KUKD | |
| Uroflowmetry | ZZ1280 | |
| Residual urine | ZZ1291 | |
| Fluid and urination schedule | ZZ1295 | |

**Sensitivity analyses**

Table S3. Cumulative incidence (%, 95% CI) of urologic cancer in patients with a first-time diagnosis of recurrent cystitis in Denmark, 2006-2022. Restricted to patients with at least one recorded UTI in the year before the recurrent cystitis diagnosis.

| | 1-year incidence | 2-year incidence | 5-year incidence |
|---|---|---|---|
| Any urologic cancer | 1.2 (1.1-1.3) | 1.4 (1.3-1.6) | 2.1 (1.9-2.2) |
| Age group | | | |
| 18 – 59 years | 0.4 (0.3-0.5) | 0.5 (0.4-0.6) | 0.7 (0.6-0.9) |
| 60 – 74 years | 1.8 (1.6-2.1) | 2.2 (1.9-2.4) | 3.3 (3.0-3.7) |
| ≥ 75 years | 1.8 (1.5-2.1) | 2.4 (2.1-2.7) | 3.2 (2.9-3.7) |
| Sex | | | |
| Women | 0.6 (0.5-0.7) | 0.7 (0.6-0.8) | 0.9 (0.8-1.0) |
| Men | 2.5 (2.2-2.8) | 3.1 (2.8-3.4) | 4.7 (4.3-5.1) |
| Any urologic cancer minus prostate cancer | 0.7 (0.6-0.8) | 0.9 (0.8-1.0) | 1.2 (1.1-1.3) |
| Men | 1.0 (0.8-1.2) | 1.3 (1.1-1.5) | 1.8 (1.5-2.0) |
| Urologic cancers | | | |
| Urinary bladder | 0.6 (0.5-0.7) | 0.7 (0.6-0.8) | 0.9 (0.8-1.0) |
| Kidney | 0.1 (0.1-0.1) | 0.1 (0.1-0.2) | 0.2 (0.2-0.3) |
| Ureter and renal pelvis | 0.0 (0.0-0.0) | 0.0 (0.0-0.1) | 0.1 (0.0-0.1) |
| Prostate[a] | 1.5 (1.3-1.7) | 1.8 (1.5-2.0) | 2.9 (2.6-3.3) |

[a] Calculated among men only

Abbreviations: CI, Confidence Interval

Table S4. Standardised incidence ratios, SIR (95% CI), of urologic cancer following a first-time diagnosis of recurrent cystitis in Denmark, 2006-2022, according to sex, age, haematuria, and follow-up time. Restricted to patients with at least one recorded UTI in the year before the recurrent cystitis diagnosis.

| | 0 - <1 year | 1 - <2 years | ≥2 years | Overall |
|---|---|---|---|---|
| All patients | 4.86 (4.42-5.32) | 1.07 (0.86-1.31) | 0.98 (0.88-1.08) | 1.61 (1.51-1.72) |
| Sex | | | | |
| Women | 11.44 (9.71-13.39) | 2.08 (1.36-3.04) | 1.52 (1.24-1.85) | 3.04 (2.70-3.42) |
| Men | 3.77 (3.36-4.21) | 0.90 (0.70-1.14) | 0.87 (0.78-0.97) | 1.35 (1.25-1.45) |
| Age groups, years | | | | |
| 18-59 | 12.92 (10.25-16.08) | 1.21 (0.52-2.39) | 1.22 (0.98-1.51) | 2.10 (1.80-2.44) |
| 60-74 | 4.63 (4.05-5.27) | 0.82 (0.58-1.12) | 0.97 (0.85-1.10) | 1.48 (1.36-1.61) |
| ≥ 75 | 3.87 (3.28-4.54) | 1.42 (1.03-1.90) | 0.80 (0.63-1.01) | 1.65 (1.46-1.86) |
| Haematuria | 6.61 (4.23-9.83) | 1.59 (0.51-3.70) | 1.10 (0.60-1.85) | 2.21 (1.60-2.97) |

*Observed number of cancers not reported according to Danish data protection rules to prevent back-calculation of cells with less than 5 observations.

Abbreviations: O, observed cancers; SIR, standardised incidence ratio; CI, confidence interval; N/A, Not applicable.

Table S5. Standardised incidence ratios, SIR (95% CI), of urologic cancers in patients with a first-time diagnosis of recurrent cystitis in Denmark, 2006-2022. Restricted to patients with at least one recorded UTI in the year before the recurrent cystitis diagnosis.

| | Women | Men | Overall |
|---|---|---|---|
| All patients | 3.04 (2.70-3.42) | 1.35 (1.25-1.45) | 1.61 (1.51-1.72) |
| Type of diagnosis | | | |
| Primary | 2.88 (2.52-3.29) | 1.19 (1.09-1.30) | 1.46 (1.35-1.57) |
| Secondary | 3.85 (2.93-4.96) | 2.12 (1.82-2.45) | 2.38 (2.09-2.70) |
| Specialty | | | |
| Urology | 3.23 (2.76-3.75) | 1.33 (1.22-1.46) | 1.57 (1.45-1.69) |
| Non-urology | 2.80 (2.31-3.37) | 1.39 (1.20-1.61) | 1.73 (1.54-1.94) |
| Comorbidities[1] | | | |
| Diabetes | 4.02 (2.84-5.52) | 1.16 (0.92-1.46) | 1.52 (1.26-1.83) |
| Hypertension | 3.56 (2.88-4.35) | 1.17 (0.99-1.37) | 1.57 (1.38-1.77) |
| Chronic kidney disease | 4.58 (2.96-6.76) | 1.48 (1.12-1.92) | 1.87 (1.48-2.32) |
| Obesity | 3.27 (2.10-4.87) | 1.66 (1.12-2.37) | 2.12 (1.60-2.77) |
| Chronic obstructive pulmonary disease | 3.40 (2.07-5.24) | 1.00 (0.67-1.44) | 1.41 (1.04-1.86) |
| Benign prostatic hyperplasia[2] | - | 1.16 (0.95-1.41) | 1.16 (0.95-1.41) |
| Gynaecological diseases[3] | 2.80 (2.09-3.67) | - | 2.80 (2.09-3.67) |
| Modified CCI score[4] | | | |
| 0 | 2.75 (2.34-3.22) | 1.38 (1.25-1.52) | 1.60 (1.47-1.74) |
| 1-2 | 3.50 (2.85-4.25) | 1.33 (1.15-1.52) | 1.66 (1.48-1.86) |
| ≥3 | 3.59 (2.28-5.39) | 1.26 (0.97-1.62) | 1.53 (1.22-1.89) |
| Number of UTI episodes[5] | | | |
| 1-2 | 2.96 (2.36-3.68) | 1.40 (1.26-1.55) | 1.55 (1.41-1.71) |
| 3-5 | 3.05 (2.56-3.60) | 1.37 (1.20-1.54) | 1.69 (1.53-1.86) |
| ≥6 | 3.14 (2.41-4.03) | 1.07 (0.82-1.38) | 1.60 (1.33-1.91) |
| Urinary tract stone[1] | 3.24 (1.77-5.44) | 1.14 (0.80-1.57) | 1.39 (1.03-1.82) |

[1] Look-back period of 10 years from recurrent cystitis diagnosis
[2] Among men only
[3] Among women only
[4] Excluding cancers
[5] Look-back period of 1 year from recurrent cystitis diagnosis
Abbreviations: O, Observed cancers; CCI, Charlson Comorbidity Index; CI, confidence interval
